# Old woman with Sheehan's syndrome suffered severe hyponatremia following percutaneous coronary intervention: a case report and review of literature

**DOI:** 10.3389/fcvm.2024.1353392

**Published:** 2024-04-29

**Authors:** Jie Gao, Yuehai Wang, Anqi Zhang, Huihui Pang, Fei Wang

**Affiliations:** ^1^ School of Clinical Medicine, Shandong Second Medical University, Weifang, Shandong, China; ^2^ Cardiology Department and Experimental Animal Center, Liaocheng People’s Hospital of Shandong University and Liaocheng Hospital Affiliated to Shandong First Medical University, Liaocheng, Shandong, China; ^3^ Department of Central Laboratory, Liaocheng People’s Hospital, Liaocheng, Shandong, China; ^4^ Department of Cardiology, Shandong Corps Hospital of Chinese People’s Armed Police Forces, Jinan, China

**Keywords:** Sheehan’s syndrome, percutaneous coronary intervention, severe hyponatremia, glucocorticoid deficiency, stress, contrast agent, coronary atherosclerotic disease

## Abstract

Glucocorticoid deficiency can lead to hypoglycemia, hypotension, and electrolyte disorders. Acute glucocorticoid deficiency under stress is very dangerous. Here, we present a case study of an elderly patient diagnosed with Sheehan's syndrome, manifesting secondary adrenal insufficiency and secondary hypothyroidism, managed with daily prednisone and levothyroxine therapy. She was admitted to our hospital due to acute non-ST segment elevation myocardial infarction. The patient developed nausea and limb twitching post-percutaneous coronary intervention, with subsequent diagnosis of hyponatremia. Despite initial intravenous sodium supplementation failed to rectify the condition, and consciousness disturbances ensued. However, administration of 50 mg hydrocortisone alongside 6.25 mg sodium chloride rapidly ameliorated symptoms and elevated blood sodium levels. Glucocorticoid deficiency emerged as the primary etiology of hyponatremia in this context, exacerbated by procedural stress during percutaneous coronary intervention. Contrast agent contributed to blood sodium dilution. Consequently, glucocorticoid supplementation emerges as imperative, emphasizing the necessity of stress-dose administration of glucocorticoid before the procedure. Consideration of shorter intervention durations and reduced contrast agent dosages may mitigate severe hyponatremia risks. Moreover, it is crucial for this patient to receive interdisciplinary endocrinologist management. In addition, Sheehan's syndrome may pose a risk for coronary atherosclerotic disease.

## Introduction

In developed countries, studies have revealed varying prevalence rates of Sheehan's syndrome (SHS) among women, ranging from 0.0051% (1) to 3.1% (2). There were also studies showing that the prevalence of SHS ranged from 1% to 2% among women who experienced hypotension due to blood loss of 1–2 L (3, 4). Contrastingly, in undeveloped nations, the prevalence varies from 3.1% to 27.6% (5–7). The diagnostic journey for SHS patients spans a considerable duration of 7–19 years from symptom onset to definitive diagnosis (8). Sheehan's syndrome arises from ischemic necrosis of the anterior pituitary gland triggered by postpartum hemorrhage (8), leading to pituitary hormone dysfunction, including insufficient secretion of growth hormone, thyroid stimulating hormone, gonadotropin, prolactin, and adrenocorticotropin (ACTH) (7, 9). Predominant symptoms are associated with dysfunction of the gonads, thyroid, and adrenal cortex due to insufficient secretion of gonadotropins, thyroid stimulating hormones, and ACTH, respectively. The latter is the most prominent and sometimes life-threatening. Supplementing various deficient hormones is the primary treatment for SHS.

Glucocorticoids, pivotal adrenal cortex hormones, play crucial roles in regulating glucose metabolism, blood pressure, and electrolyte balance. Deficiency in glucocorticoids can lead to hypoglycemia, hypotension, and electrolyte disturbances. Lifetime glucocorticoid replacement therapy stands as a cornerstone in managing SHS patients. Fluctuations in neuroendocrine system activity necessitate adjustments in glucocorticoid supplementation, while metabolic disruptions from other etiologies also dictate dosage alterations. Inadequate comprehension of these dynamics among healthcare professionals may impact the prognosis of SHS patients and predispose them to risks. Surgical treatments, including interventional procedures, represent significant stressors in medical care. Failure to administer preoperative stress doses of glucocorticoids to SHS patients can engender serious consequences. To our knowledge, this article represents the first documented case of severe hyponatremia in an SHS patient following percutaneous coronary intervention (PCI).

## Case presentation

**Figure 1:**
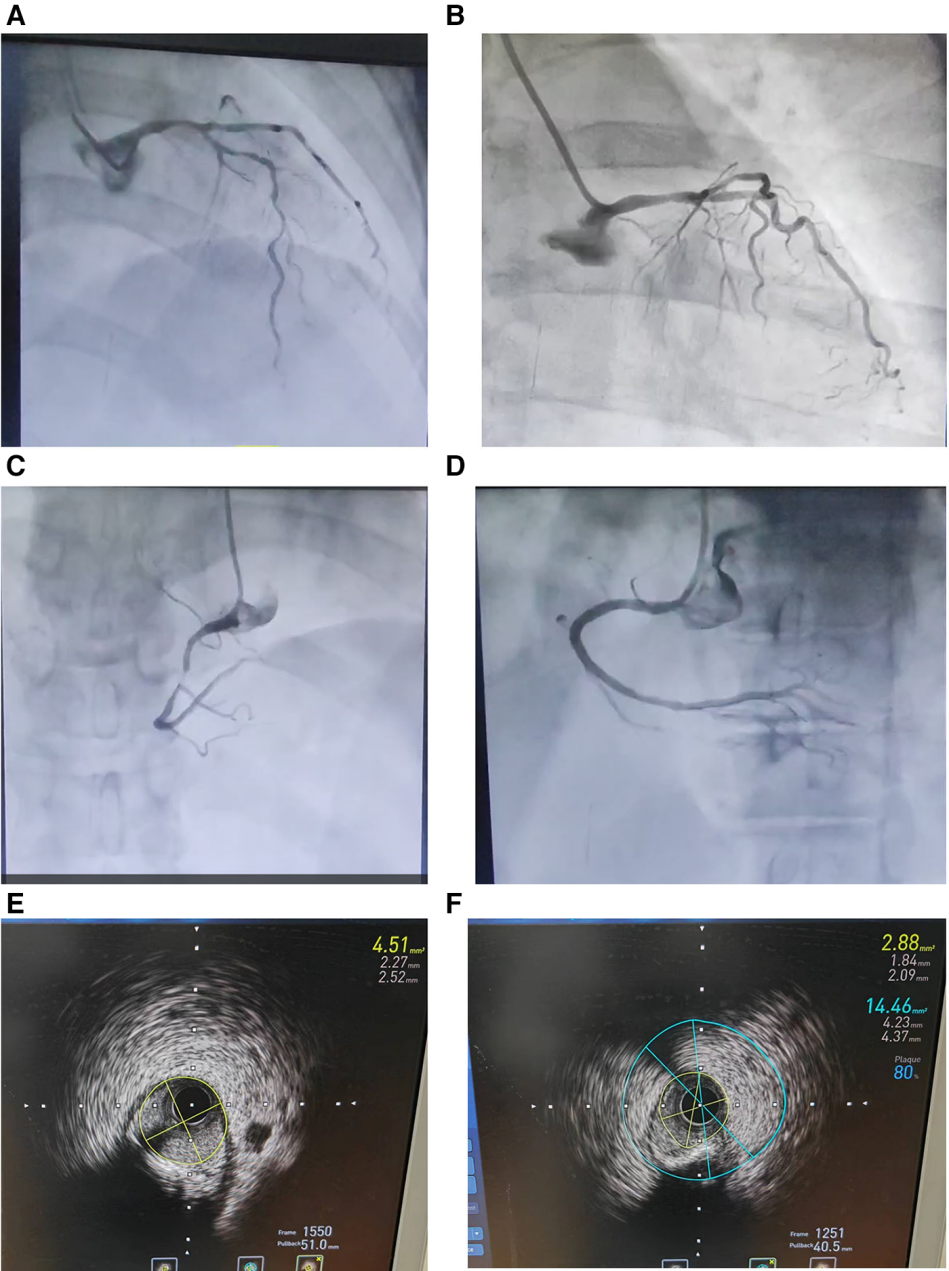
Coronary angiography (**A**–**D**) and intravascular ultrasound examination (**E and F**) in an elderly patient with Sheehan's syndrome. (**A**) The stenosis degree is 40%, 80%, and 60%, respectively, at the end of the left main trunk, the proximal and middle segments of the left anterior descending branch. (**B**) The stenosis degree is 98% at the proximal segments of the left circumflex branch. (**C**) The stenosis degree is almost completely occluded at the middle segment of the right coronary artery. (**D**) Two stents are inserted in the middle segment of the RCA. (**E**) The minimum lumen area at the distal stenosis of the left main trunk is 4.51 mm^2^. (**F**) The plaque load at the most severe stenosis of the proximal left anterior descending branch is 80%, and the minimum lumen area is 2.88 mm^2^.

A 70-year-old female patient presented with paroxysmal exertional chest tightness persisting for one month, alleviated by a few minutes of rest. Forty years ago, the patient suffered from postpartum hemorrhage, without blood transfusion, subsequently developing lactation failure and amenorrhea. Five years later, she was diagnosed with SHS at the Affiliated Hospital of Shandong University. Management included 5 mg of prednisone acetate in the morning for secondary adrenal insufficiency, and 50 ug of levothyroxine for secondary hypothyroidism. Apart from medication adherence, the patient lacked awareness regarding adrenal insufficiency. The patient had a decade-long history of hypertension, controlled with 5 mg of telmisartan and 5 mg of amlodipine daily. This patient had a weight of 46 kl, a height of 1.57 m, and a BMI of 18.66 kg/m^2^. Upon hospital admission, her vital signs were stable with a blood pressure of 122/58 mmHg, and a heart rate of 65 beats per minute. Physical examination revealed no pulmonary rales, cardiac murmurs, lower limb edema. Laboratory finding indicated elevated blood troponin I (0.5487 ng/ml, 0–0.0175 ng/ml), normal blood sodium (141.5 mmol/L, 137 mmol/L–147 mmol/L), and elevated fasting total cholesterol (6.28 mmol/L, 3 mmol/L–5.7 mmol/L). Thyroid function tests revealed low level of free thyroxine (FT4) (6.77 pmol/L, 7.98 pmol/L–16.02 pmol/L), with normal levels of free triiodothyronine (FT3) and thyroid stimulating hormone. Electrocardiogram indicated sinus bradycardia. We diagnosed the patient with acute non-ST segment elevation myocardial infarction (NSTEMI) and performed percutaneous coronary angiography (CAG) and intravascular ultrasound (IVUS) examination. We found that the stenosis degree was 40%, 80%, and 60%, 98%, and almost completely occluded, respectively, in the left main trunk (LM), the proximal and middle segments of the left anterior descending branch (LAD), the proximal segments of the left circumflex branch (LCX), and the middle segment of the right coronary artery (RCA) (Figures 1A–C). The minimum lumen area at the distal stenosis of the LM was 4.51 mm^2^ (Figure 1E), the plaque load at the most severe stenosis of the proximal LAD was 80%, with a minimum lumen area of 2.88 mm^2^ (Figure 1F). Due to the patient's refusal to undergo coronary artery bypass grafting, two stents were inserted in the middle segment of the RCA (Figure 1D). The intervention lasted for 2 h, including coronary angiography, bilateral intravascular ultrasound examination, patient involvement in treatment decision-making based on examination results, and subsequent coronary intervention treatment, utilizing 130 ml of iodixanol. The patient did not experience any chest discomfort, but was nervous and had a blood pressure rise to 190/100 mmHg, managed with sublingual nifedipine tablets and intravenous isosorbide nitrate. Following percutaneous intervention (PCI), the patient experienced a sequence of symptoms from the 12th to the 50th h, including nausea and loss of appetite, profuse sweating, mild limb twitching, and drowsiness in sequence (Table 1). Limb twitching persisited for 18 h from the 38th to the 56th h post-PCI. On the 24th h post-PCI, the patient was diagnosed with hyponatremia (Table 1), and 2%−3% sodium chloride was intermittently administered intravenously. Despite increased sodium chloride supplementation, symptoms persisted until administration of hydrocortisone, leading to symptom resolution and rapid improvement in blood sodium levels (Table 1). By the 62nd h post-PCI, symptoms of hyponatremia completely resolved, with blood sodium level increasing from 114.2 mmol/L to 132 mmol/L (Table 1). At the 86th h post-PCI, blood sodium level returned to normal. After 40 h, blood tests revealed low levels of cortisol (2.76 ug/dl, 6.7ug/dl–22.6 ug/dl), ACTH (4.26 pg/ml, 10.1 pg/ml–57.6 pg/ml), FT3 (3.41 pmol/L, 3.53 pmol/L−7.37 pmol/L), and FT4 (7.12 pmol/L, 7.98 pmol/L–16.02 pmol/L). Following discharge, the patient continued oral medication with 2.5 mg prednisone acetate and 50 ug levothyroxine sodium daily, as well as dual antiplatelet drugs, statins, and antihypertensive agents. During the next nine-month follow-up period, the patient did not experience ischemic symptoms or hyponatremia.

**Table 1:** Timeline of changes in symptoms, blood sodium titers, and hyponatremia treatment in this patient at 12, 24, 38, 50, 56, 62 and 86 h after percutaneous intervention. normal titer blood sodium reference value: 137 mmol/L to 147 mmol/L.

	Pre-operative	Post-operative						
		12 h	24 h	38 h	50 h	56 h	62 h	86 h
Nausea		Yes	Yes	Yes	Yes			
Poor appetite		Yes	Yes	Yes	Yes	Yes		
Sweating			Yes	Yes	Yes			
Limb twitching				Yes	Yes			
Drowsiness					Yes			
Blood sodium titers (mmol/L)	141.5		117	109.7	114.2		132	136
Intravenous treatment with sodium chloride (g)		4.5	6.5	17.55	6.25		10.75	
Intravenous treatment with hydroprednisone (mg)				10				
Intravenous treatment with hydrocortisone (mg)					50			

## Discussion

### SHS and hyponatremia

Sheehan's syndrome is characterized by insufficient secretion of ACTH due to pituitary necrosis, resulting in decreased synthesis and secretion of adrenocortical hormones, particularly glucocorticoids. Glucocorticoids play a vital role in regulating sodium and water excretion and maintaining electrolyte balance in the body. Insufficient glucocorticoid levels lead to diminished renal free water clearance, causing water retention and dilutional hyponatremia, resulting in reduced plasma osmolality. Furthermore, despite low osmolality, there is inappropriate secretion of antidiuretic hormone (vasopressin) due to the absence of cortisol's tonic inhibition (10).

### Clinical presentation and management

In this case, the patient had a medical history of a SHS diagnosis, presenting with secondary adrenal insufficiency and secondary thyrotrophin deficiency necessitating hormone replacement therapy. Secondary adrenal insufficiency arises from pituitary impairment, causing decreased production of ACTH and subsequent reduction in adrenal stimulation, leading to decreased cortisol production. Glucocorticoid deficiency emerged as the primary mechanism of hyponatremia in this patient. During the 2-h of coronary diagnosis and treatment, the patient was anxious, had high blood pressure, and was in a severe stress state, which required additional cortisol to cope with. The specific amount could be evaluated by a specialist doctor. However, due to secondary adrenal insufficiency, the patient could not suddenly increase the secretion of glucocorticoids to copy with the stress. Additionally, glucocorticoids were not pre increased before the procedure. Therefore, the patient was at risk of acute and severe adrenal cortical hormone deficiency, leading to excessive sodium loss, water retention, and subsequent hyponatremia.

### Treatment response

Despite intravenous supplementation of 24.05 g sodium chloride within 26 h, hyponatremia persisted, accompanied limb twitching and drowsiness, indicating an exacerbation of hyponatremia and the formation of hypotonic brain edema. Administration of 50 mg hydrocortisone effectively relieved excessive sodium excretion and water retention. Even with 6.25 g sodium chloride treatment, the patient's symptoms almost disappeared after 6 h, and blood sodium increased from 114.2 mmol/L to 132 mmol/L after 12 h. The subsequent increase in blood sodium levels highlights the importance of glucocorticoid replacement therapy in managing hyponatremia secondary to SHS.

### Management considerations

The case underscores the importance of preoperative stress dose glucocorticoid therapy in SHS patients undergoing procedures such as PCI. However, we were unaware the importance. Additionally, awareness of the potential for contrast agents to induce dilutional hyponatremia and stress response caused by PCI is crucial. Lack of endocrinologist consultation before the procedure and inadequate patient education regarding adrenal insufficiency contributed to the suboptimal management of this patient. Inappropriately administered sublingual nifedipine treatment, intended to manage transient hypertension, not only increased the risk of acute cardiovascular and cerebrovascular disease, but also increased the risks of further activating the sympathetic nervous (11) and exacerbating stress. Therefore, the interdisciplinary management involving endocrinologists is crucial for optimizing the treatment for patients with complex endocrine disorders like SHS, facilitating appropriate examinations, treatment and health education to prevent adrenal crisis and improve long-term outcomes (12, 13).

### Prolonged limb twitching and sodium correction

Unlike the transient symptoms of epilepsy, the patient experienced persistent limb twitching for up to 18 h, possibly due to prolonged lower blood sodium levels. This prolonged imbalance could have led to sustained electrical instability in brain cells, resulting in repetitive abnormal electro-discharge and impaired brain function, posing significant risks to the patient. However, our approach to correcting hyponatremia may not have followed optimal guidelines. Our method of correcting hyponatremia may not have followed the best guidelines. The target value for increasing serum sodium was not set to not exceed 8–10 mmol/L/24 h (14). Our treatment rapidly increased the patient's blood sodium from 114 mmol/L to 132 mmol/L in 12 h, and then continued to supplement with hypertonic sodium chloride. Within 26 h after identifying hyponatremia, 24.05 g of sodium chloride was administered intravenously. These treatments are unreasonable, and the overly rapid correction of hyponatremia may be a risk factor for osmotic demyelination syndrome. Proper management should aim to increase blood sodium concentration gradually, with close monitoring to prevent such complications.

### Other proposed mechanisms of hyponatremia

Contrast agents have been implicated in inducing hyponatremia, particularly in women (15–18). Following administration, the contrast agents elevate the osmotic pressure of extracellular fluid, leading to passive water transfer of intracellular to extracellular compartments and resultant diluted hyponatremia (15, 16). Sweating caused by sympathetic nerve stimulation and sweating caused by adverse reactions to iodixanol injection may also contribute to sodium loss.

### Role of hypothyroidism

The patient's thyroid hormone levels were low before and after the procedure, indicating the presence of secondary hypothyroidism. Hypothyroidism may have contributed to hyponatremia mainly through the reduced ability to excretal free water, caused by higher levels of ADH. The elevation in ADH levels is largely due to the decrease in cardiac output that stimulates the carotid sinus baroreceptors, prompting the release of ADH. In addition, hypothyroidism can promote hyaluronic acid deposition in extravascular tissues, leading to increased water retention and reduced blood volume. This not only reduces glomerular filtration, but also increases the secretion of antidiuretic hormone, thereby increasing the risk of diluted hyponatremia (19–22). Therefore, optimizing levothyroxine therapy to restore normal thyroid hormone levels may help mitigate the risk of hyponatremia in such cases.

### SHS and coronary artery disease

Previous studies have indicated a higher mortality rate in patients with pituitary dysfunction, primarily attributed to cardiovascular diseases (23–25). Due to chronic inflammation, dyslipidemia, and abdominal obesity, patients with SHS tend to develop coronary artery disease (CAD) (26). This NSTEMI patient suffered from severe coronary atherosclerosis, with traditional risk factors including hypertension and hypercholesterolemia. Long-term oral administration of glucocorticoids may be associated with hypertension and hyperlipidemia in such patients (27, 28). In addition, hypothyroidism, which is common in SHS, can also contribute to hyperlipidemia (29).

Although severe hyponatremia following PCI in SHS patients is not extensively reported, there are cases of female patients exhibiting life-threatening adrenal dysfunction post-PCI (30, 31). The lowest blood sodium level in these cases is 122 mmol/L, and there is no hypoglycemia. Glucocorticoids have good therapeutic effects. The difference is that these patients exhibit significant hypotension, shock, and even Takotsubo syndrome (30, 31).

## Conclusions

The deficiency of glucocorticoids caused by secondary adrenal insufficiency is the primary mechanism for severe hyponatremia in this patient with SHS. The stress induced by PCI exacerbates glucocorticoid deficiency. The contrast agent further contributes to dilutional hyponatremia. The preoperative stress dose of glucocorticoid is crucial to avoid this complication. Glucocorticoids were crucial in correcting severe hyponatremia in this SHS patient with secondary adrenal insufficiency. Shortening the duration of PCI and minimizing the dosage of contrast agents may be beneficial for preventing severe hyponatremia. Meanwhile, it is also crucial for this SHS patient to receive interdisciplinary management involving endocrinologists before and after the procedure. Additionally, SHS may serve as a potential risk factor for CAD.

## Data Availability

The original contributions presented in the study are included in the article/Supplementary Material, further inquiries can be directed to the corresponding author.
